# Muscle-Specific PPARβ/δ Agonism May Provide Synergistic Benefits with Life Style Modifications

**DOI:** 10.1155/2007/30578

**Published:** 2007-12-12

**Authors:** Adnan Erol

**Affiliations:** Department of Internal Medicine, Faculty of Medicine, Celal Bayar University, Manisa 45040, Turkey

## Abstract

Peroxisome proliferator-activated receptor β/δ (PPARβ/δ) has emerged as a powerful metabolic regulator in diverse tissues including fat, skeletal muscle, and the heart. It is now established that activation of PPARβ/δ promotes fatty acid oxidation in several tissues, such as skeletal muscle and adipose tissue. In muscle, PPARβ/δ appears to act as a central regulator of fatty acid catabolism. PPARβ/δ contents are increased in muscle during physiological situations such as physical exercise or long-term fasting, characterized by increased fatty acid oxidation. Targeted expression of an activated form of PPARβ/δ in skeletal muscle induces a switch to form increased numbers of type I muscle fibers resembling the fiber type transition by endurance training. Activation of PPARβ/δ also enhances mitochondrial capacity and fat oxidation in the skeletal muscle that resembles the effect of regular exercise. Therefore, it is hypothesized that muscle-specific PPARβ/δ agonists could be a key strategy to support the poor cardiorespiratory fitness associated with metabolic disorders.

## 1. INTRODUCTION

Peroxisome proliferator-activated receptor *β*/*δ* (PPAR*β*/*δ*) has emerged as a powerful metabolic regulator in diverse tissues including fat, skeletal muscle, and the heart. It is now established that activation of PPAR*β*/*δ* promotes fatty acid oxidation in several tissues, such as skeletal muscle and adipose tissue. In muscle, PPAR*β*/*δ* appears to act as a central regulator of fatty acid catabolism. PPAR*β*/*δ* contents are increased in muscle during physiological situations such as physical exercise or long-term fasting, characterized by increased fatty acid oxidation [1]. Targeted expression of an activated form of PPAR*β*/*δ* in skeletal muscle induces a switch to form increased numbers of type I muscle fibers resembling the fiber type transition by endurance training [2]. Activation of PPAR*β*/*δ* also enhances mitochondrial capacity and fat oxidation in the skeletal muscle that resembles the effect of regular exercise [2]. These raise a parallel question of whether PPAR*β*/*δ* agonists, as with constitutive genetic activation of PPAR*β*/*δ* in skeletal muscle, can drive the formation of oxidative myofibers and enhance physical activity endurance [3].

The beneficial effect of exercise on cardiovascular fitness has proven particularly successful as a treatment for the metabolic diseases, including type 2 diabetes [4]. Regular exercise has been shown to induce changes in both skeletal muscle metabolism and muscle fiber type over time, most notably an increase in mitochondrial content and oxidative metabolism as well as a shift toward a more slow oxidative fiber type [5]. Exercise-related adjustments in fuel homeostasis and fiber type changes are mediated not only by an increase in mitochondrial number but also by functional changes in the resident mitochondrial population [6]. Six weeks of running endurance training in human skeletal muscle reasoned in a shift toward an increased type I muscle fiber phenotype, which supports the results of mice experiments [7].

Skeletal muscle is a major player in glucose homeostasis under basal conditions and in response to insulin and exercise. Therefore, skeletal muscle must be considered an important therapeutic target tissue in the battle against cardiovascular disease. Cardiovascular risk factors are directly influenced by diet, metabolism, and physical activity. Metabolism and physical activity, in turn, are primarily driven by skeletal muscle [8]. However, the type of fuels, style of activity, and neuronal innervations affect muscle energy metabolism. In fact, muscle mass is not the only determinant of muscle function, and aerobic exercise training may import positive effects on neuromuscular adaptations and, consequently, muscle quality especially in individuals who were sedentary and sarcopenic prior to the exercise intervention [9]. Emerging evidence suggests that skeletal muscle stimulated changes in energy homeostasis reason in gene expression and contribute to muscle plasticity. A number of energy-sensing molecules have been shown to sense the variations in energy homeostasis. These molecules may therefore sense information relating to the intensity, duration, and the frequency of muscle exercise [10]. Muscular activity acts as a powerful stimulus for the hypothalamic-pituitary axis, leading to the liberation of several neuroendocrine hormones, which are accurate regulators of fuel homeostasis [11].

Human skeletal muscle may appear to be homogenous, but in fact it is composed of distinct fiber types, referred as slow and fast, defined by the myosin isotype expressed in the particular fiber. Slow muscle fiber expresses type I myosin; fast fibers can express types IIa, IIb, and IIx. The variety in fiber type enables the person to perform different types of work. Given the high degree of skeletal muscle plasticity in humans with exercise, it is likely that the contraction function of slow-twitch and fast-twitch muscle fibers undergo differential alterations with distance running training [8]. Interventions including endurance exercise, physical inactivity, and metabolic diseases such as type 2 diabetes mellitus can induce the transdifferentiation of myofibers [12].

Skeletal muscle fibers are classified by two major functional characteristics: speed of contraction, and the aerobic (oxidative)/anaerobic (glycolytic) production of ATP. The speed of the fiber reflects how fast the fiber hydrolyses ATP [13]. Fast fibers (type IIb or white fibers) utilize anaerobic glycolysis, are low in mitochondria and myoglobin and rich in glycogen, and are suited to short-term intense activity. Slow fibers (type I or red fibers) utilize aerobic metabolism and are suited to endurance activity. There is an intermediate fast fibers type IIa that combine fast-twitch capacity with aerobic fatigue resistant metabolism and intermediate glycogen levels [13]. In general, the metabolic characteristics of type IIb fibers include a reduced oxidative enzyme activity and an increased glycolytic enzyme activity in comparison with type I muscle fibers (oxidative slow-twitch) or type IIa (oxidative fast-twitch) [14]. Moreover, it has been postulated, mostly based on animal studies, that muscle fibers follow an order of type I > type IIa > type IIb for insulin sensitivity [15].

One of the genes induced in skeletal muscle after exercise is the peroxisome proliferator-activated receptor (PPAR)-*γ* coactivator-1*α* (PGC-1*α*). PGC-1*α*, a coactivator of several nuclear receptors and other transcription factors, has been shown to be involved in the regulation of mitochondrial biogenesis, adaptive thermogenesis, and enzymes involved in fatty acid oxidation [16]. It is now well established that PGC1*α* induces a remodeling of skeletal muscle fiber composition toward more oxidative type I fibers. The expression of PGC1*α* in skeletal muscle is readily inducible by both short-term exercise and endurance training in animal models and human [17]. In human skeletal muscle, endurance training induces an increase in PGC1*α*, particularly in type IIa fibers. This means that when type IIa fibers are properly activated, they promote mitochondrial biogenesis and the switch of muscles to a type I phenotype [7]. However, PGC1*α* has been shown to inhibit the insulin signaling pathway in the liver, and increased hepatic PGC1*α* expression could be expected to stimulate hepatic glucose output contributing to the hyperglycemic state [18]. Furthermore, PGC1*α* expression has been reported to be increased in liver of both type 1 and type 2 diabetic mouse models [19].

As mentioned above, PGC1*α* is a master regulator of mitochondrial biogenesis. Peroxisome proliferator response element (PPRE) in the distal region of PGC1*α* binds PPAR*β*/*δ*. Consequently, activation of PPAR*β*/*δ*, but not PPAR*α*, induces transcription of the PGC1*α* gene in muscle [20]. In contrast, it has been reported that in transgenic mice overexpressing PPAR*β*/*δ* [2, 21], and in rats in which PPAR*β*/*δ* activity is increased by raising plasma fatty acids [22], mitochondrial biogenesis is augmented without an increase in PGC1*α* expression.

Still unanswered question is whether PGC1*α* activation alone, in the absence of exercise, would be sufficient to confer protection against diabetes or not. Although muscle-specific overexpresion of PGC1*α* in transgenic mice results in mitochondrial proliferation and increased expression of genes involved in oxidative phosphorylation, the impact of this manipulation on whole body glucose homeostasis has not been reported [5].

## 2. PPARs: LIPID SENSORS AND TRANSCRIPTIONAL SWITCHES

Metabolism, in part, is regulated by nuclear receptors. Essentially, these receptors function as the conduit between environmental stimuli and gene expression, and mediate the physiological response [23]. PPARs are a subgroup of the nuclear receptor superfamily of ligand-inducible transcription factors [23]. They form heterodimers with retinoid X receptors (RXRs) and bind to consensus DNA sites. In the absence of ligand, PPAR-RXR heterodimers recruit corepressors and associated some other modifying factors and silence transcription. Ligand binding induces a conformational change in PPAR-RXR complexes, releasing repressors in exchange for coactivators. Unlike classical endocrine receptors that bind to high-affinity glandular hormones, ligand-activated PPARs turn on feed-forward metabolic cascades to regulate lipid homeostasis via the transcription of genes involved in lipid metabolism, storage, and transport. Additionally, PPARs may suppress inflammation through the stabilization of repressive complexes at inflammatory gene promoters [3].

Three PPAR isotypes, *α*, *β* or *δ*, and *γ*, have been determined so far in mammalian. PPARs act as nutritional lipid sensors and control transcriptional rate of a large panel of genes implicated in organogenesis, cell proliferation, cell differentiation, inflammation, and metabolism of lipid or carbohydrates [24, 25]. PPAR*α* and PPAR*γ* are the most extensively examined because they are involved in the effects of marketed compounds with pharmaceutical interest [25].

Fatty acid catabolism is very active in muscle and utilization of lipids is enhanced in physiological situations such as fasting and physical exercise [25]. PPAR*α* is known regulator of fatty acid oxidation gene expression. PPAR*α*-specific agonists stimulate mitochondrial *β*-oxidation. However, PPAR*α* knock-out mice exhibited minimal alteration in skeletal muscle fatty acid oxidative capacity [26]. Several lines of evidence have established that PPAR*β*/*δ*, which is the predominant isotype expressed in skeletal muscle, plays a central role in the control of lipid metabolism of this tissue [1, 25]. This high abundance of PPAR*β*/*δ* may compensate for the lack of PPAR*α* in the knock-out mice [25, 26].

Clear improvements in skeletal muscle metabolism mediated by nuclear receptor function and pharmacological activation will promote improved carbohydrate and lipid metabolism in skeletal muscle because numerous metabolic genes regulated by nuclear receptors have been identified ranging from transport molecules, enzymes involves in lipid and carbohydrate metabolism, lipid and carbohydrate storage, thermogenesis, and signaling pathways [23, 24]. Hence, skeletal muscle has a paramount role in energy balance, and is the primary tissue of insulin-stimulated glucose uptake, disposal, and storage, regulates cholesterol efflux, and strongly influences metabolism via modulation of circulating and stored lipid flux. For example, lipid catabolism supplies up to 70% of the energy requirements for resting muscle [13].

PPAR*β*/*δ* agonists have a significant role in the regulation of the mRNAs encoding the uncoupling proteins (UCPs), mitochondrial proton carriers, which control metabolic efficiency, energy expenditure, and adaptation to nutrient (i.e., preferential lipid utilization and thermogenesis by uncoupling oxidation/respiration from ATP synthesis) [27].

## 3. IMPORTANCE OF PPAR*β*/*δ* IN MUSCLE

Unlike in liver and heart, PPAR*β*/*δ* is expressed in skeletal muscle at 10- and 50-fold higher levels compared with PPAR*α* and PPAR*γ*, respectively, and it is preferentially found in oxidative rather than glycolytic myofibers [28].

Mice in which PPAR*β*/*δ* is selectively ablated in skeletal muscle myocytes exhibited a muscle fiber-type switching toward lower oxidative capacity that preceded the development of obesity and diabetes, thus demonstrating that PPAR*β*/*δ* is instrumental in myocytes to maintenance of oxidative fiber-type switching is likely to be the cause and not the consequence of these metabolic disorders. As mentioned previously, the effect of PPAR*β*/*δ* on the formation and/or maintenance of slow muscle fibers can be ascribed, at least in part, to the stimulation of PGC1*α* expression at the transcriptional level [29].

How might endogenous PPAR*β*/*δ* become activated naturally by exercise training? It is possible that exercise generates or increases endogenous ligands for PPAR*β*/*δ*. Exercise-induced abundance of fatty acids and their metabolites can activate PPAR*β*/*δ*. In addition, exercise may stimulate expression of PGC1*α* and thereby activate PPAR*β*/*δ*. PGC1*α* physically associates with PPAR*β*/*δ* in muscle tissue and can powerfully activate it even in the absence of ligands [30].

With aging, two conceptually different kinds of muscular atrophy, acute and chronic, can occur. Acute atrophy is associated with disuse; and chronic atrophy is more typically associated with aging [31]. The number of muscle fibers of both types I and II decreased significantly after 60 years of age. Decrease in weight of the muscle with age was slight and not significant, being considered to be due to the increase in size of type I fibers after 60 years of age. The loss of fibers begins early, at 25 years, and thereafter accelerates. Total volume of muscle fibers of type I did not decrease with age. Age-related loss of skeletal muscle is associated with a selective atrophy of the type II fibers [32].

The greater age-related mitochondrial dysfunction in muscles with high type II content provides insight into the preferential loss of type II fibers with age [33]. The tempo of mitochondrial dysfunction varies among muscles and in proportion to type II muscle fiber content, suggesting that intracellular factors, rather than time alone, may play an important role in mitochondrial aging. These defects have important impact on cell fate resulting in sarcopenia, which is a leading cause of disability in the elderly. Mitochondrial dysfunction may be an inevitable part of aging; however, in contrast to the conventional thinking that the defects are permanent, research outcomes show that at least part of this dysfunction is reversible with endurance training in human muscle [34].

Skeletal muscle must perform different kind of work, and distinct fiber types have evolved to accommodate these activities [35]. PGC1*β*, the structural homologue closest to PGC1*α*, is encoded by a separate gene and displays a similar tissue distribution. The role played by PGC1*β* is not well understood. However, PGC1*β* expression was found to be related to fat oxidation and nonoxidative glucose metabolism. Insulin increases and aging reduces skeletal muscle PGC1*α* and PGC1*β* levels. PGC1*β* expression is reduced in muscle of healthy elderly individuals and in patients with type 2 diabetes [36]. Transgenic expression of PGC1*β* causes a marked induction of type IIx fibers, which are oxidative but have “fast-twitch” biophysical properties [35]. In contrast to PGC1*α*, PGC1*β* seems not to activate nuclear receptors [37]. Therefore, pharmacologic activation of PGC1*β* is expected to decrease the age-associated progressive fast-twitch atrophy in addition to its antiobese and antidiabetic functions.

A reduced oxidative enzyme capacity of skeletal muscle has been found in type 2 diabetes as well as in obesity that is not complicated by diabetes. Although a full explanation for these differences in glycolytic and oxidative enzyme activities in skeletal muscle in obesity and type 2 diabetes compared with lean individuals has not been determined, one possibility is an altered proportion of muscle fiber types [38]. An increased proportion of type IIb muscle fibers, also termed glycolytic fast-twitch fibers, has been reported in type 2 diabetes in several studies [15]. In a recent study, in the whole muscle, oxidative activity was decreased in patients with type 2 diabetes. The slow oxidative fiber fraction was reduced by 16%, whereas the fast glycolytic fiber fraction was increased by 49% in skeletal muscle from the diabetic patients [38].

Recent evidence has demonstrated activation of PPAR*β*/*δ* in the major mass peripheral tissue (i.e., adipose and the skeletal muscle). It enhances glucose tolerance, insulin-stimulated glucose disposal, lipid catabolism, energy expenditure, cholesterol efflux, and oxygen consumption. These effects positively influence the blood-lipid profile. Furthermore, PPAR*β*/*δ*-activated type I muscle fiber abundance leads to increased endurance, insulin sensitivity, and resistance to obesity. Thus PPAR*β*/*δ* has rapidly emerged as a potential target in the battle against dyslipidemia, insulin resistance, type 2 diabetes, and obesity with therapeutic efficacy in the treatment of cardiovascular disease risk factors [39].

In addition to effects on muscle composition and metabolic capability, muscle-specific PPAR*β*/*δ* overexpression also affects adipose tissue mass. Most probably, the metabolic flux toward muscle is increasing in mice overexpressing PPAR*β*/*δ*, reducing fatty acid supply for triglyceride storage in adipose tissue. Muscle-specific PPAR*β*/*δ* overexpression promoted a shift toward smaller adipocyte size. Such a difference in cell size could account for the secretion of more adipokines such as adiponectin increase insulin sensitivity [21].

Metabolic and fiber type regulation of skeletal muscle by PPAR*β*/*δ* has several physiological implications. First, the presence of an increased proportion of oxidative slow-twitch fibers is predicted to decrease skeletal muscle fatigability. Increased endurance in marathon runners is linked to a higher proportion of oxidative slow-twitch fibers in their skeletal muscles. Second, oxidative fibers have a tremendous impact on fatty acid homeostasis. As it was clearly explained above, both obesity and insulin resistance are linked to a decrease in the proportion of oxidative slow-twitch fibers in skeletal muscle [3].

## 4. PPAR*β*/*δ* ACTION IN CARDIOMYOCYTES

In contrast to PPAR*γ* ligands where some controversy exists, administration of PPAR*β*/*δ* ligands is thought to be protective against cardiomyopathy. Impaired fatty acid oxidation and a shift to reliance on glucose metabolism are hallmarks of myocardial diseases such as cardiac hypertrophy and congestive heart failure. It was shown that cardiomyocyte-specific deletion of PPAR*β*/*δ* suppresses the expression of oxidative genes leading to impaired fatty acid oxidation and reciprocal increase in glucose oxidation, along with fat accumulation in cardiomyocytes. These alterations lead to progressive myocardial lipid accumulation, cardiac hypertrophy, and congestive heart failure with reduced survival [40]. In cultured neonatal rat ventricular cardiomyocytes, selective PPAR*β*/*δ* activation inhibits phenylephrine-induced cardiomyocyte hypertrophy and lipopolysaccharide-induced nuclear factor (NF)-*κ*B activation that may be the underlying mechanism responsible for the inhibition of cardiomyocyte growth [41].

PPAR*β*/*δ*-dependent maintenance of basal fatty acid oxidation is crucial for normal cardiac mechanics. Indeed, mice with cardiac-specific deletion of PPAR*β*/*δ* develop age-dependent cardiac lipotoxicity, cardiac hypertrophy, end-stage dilated cardiomyopathy, and decreased survival [3].

## 5. PPAR*β*/*δ* AND METABOLIC SYNDROME

It was suggested that the physiological role of PPAR*β*/*δ* may be a direct switch from glucose metabolism to fatty acid metabolism. It is conceivable that free fatty acids released from adipose tissues on fasting or exercise provide PPAR*β*/*δ* ligands to stimulate fatty acid oxidation and thermogenesis in skeletal muscle [42]. Furthermore, transgenic expression of activated PPAR*β*/*δ* in adipocytes leads to a lean phenotype and prevents high-fat diet-induced obesity in mice by increasing energy expenditure and fat oxidation. These effects appear to be due to increased thermogenesis and fat oxidation as a result of induction of UCP1 expression, and increased expression of mitochondrial enzymes of fatty acid oxidation in white adipose tissue [30].

Recent studies have reported that activation of PPAR*β*/*δ* alleviates dyslipidemia, hyperglycemia, and insulin resistance in animal models of obesity and type 2 diabetes [43, 44]. Furthermore, PPAR*β*/*δ* agonist treatment prevented weight gain, and decreased levels of serum glucose, insulin, and lipids in rats fed a high-fat diet. In addition, PPAR*β*/*δ* agonist increased expression of visfatin, adiponectin, and decreased resistin expression in both rats fed a high-fat diet and cultured 3T3-L1 adipocytes [44].

PPAR*β*/*δ* is involved in the regulation of genes participating in lipid and lipoprotein metabolism as well as in adipose tissue and muscle fatty acid oxidation. However, an increase in HDL-cholesterol is the predominant consequence of PPAR*β*/*δ* activation [45].

These data provide evidence that the activation of PPAR*β*/*δ* have an independent and additive impact on the effectiveness of aerobic physical exercise and insulin sensitivity. Provided that a number of potential adverse side-effects could be managed, high-affinity PPAR*β*/*δ* synthetic ligands would clearly be useful drugs of the future to effectively target some of the most important abnormalities associated with the metabolic syndrome such as insulin resistance, hyperglycemia, and dyslipidemia [46].

## 6. ADVERSE EFFECTS OF PPAR*β*/*δ* ACTIVATION

PPAR*β*/*δ* is a versatile regulator of distinct biological processes including and extending beyond lipid metabolism. More debatable issue is that whether PPAR*β*/*δ* is a potential regulator of adipocyte differentiation. In adult mice it comprises a nonautonomous determinant of adiposity, providing a plausible link to lipid metabolism. Apart from metabolism, PPAR*β*/*δ* was proposed to be a critical mediator of embryo implantation. During early development the receptor regulates placentation and is consequently essential for the survival of most embryos [47].

PPAR*β*/*δ* has recently been implicated in hepatic stellate cell proliferation and liver fibrosis. Hepatic stellate cells become activated in response to liver toxicants, leading to deposition of extracellular matrix and fibrosis. Ligand activation of PPAR*β*/*δ* enhanced the hepatic stellate cell proliferation and increased the synthesis of genes associated with the extracellular matrix leading to hepatic fibrosis [48].

Finally, PPAR*β*/*δ* was ascribed an oncogenic function after being identified as a direct transcriptional target of *β*-catenin. Some studies suggest that activation of PPAR*β*/*δ* is causally associated with polyp formation [49], and that increased PPAR*β*/*δ* expression is required to modulate target genes that regulate the proliferation of colon tumor cells [50]. However, current studies clearly show that specific ligand activation of PPAR*β*/*δ* leads to the induction of target gene expression associated with terminal differentiation of colonocytes. In contrast to previous reports, PPAR*β*/*δ* attenuates colon carcinogenesis [51, 52]. Thus, considerable controversy remains regarding the role of PPAR*β*/*δ* in colon cancer since there is evidence suggesting that PPAR*β*/*δ* ligands could have either positive, negative, or a combination of both effects on colon carcinogenesis.

Activation of PPAR*β*/*δ* by an agonist ligand can result in increased proliferation of breast and prostate cancer cell lines, as well as endothelial cells, and supports the hypothesis that PPAR*β*/*δ* antagonists might be of therapeutic value in the management of common epithelial cancers [53].

There is also evidence that PPAR*β*/*δ* ligands may influence skin carcinogenesis. Ligand activation of PPAR*β*/*δ* induces terminal differentiation and an apoptotic-like pathway in keratinocytes, along with inhibition of cell proliferation [44]. In addition, PPAR*β*/*δ* induces cyclooxygenase-2 (COX-2) expression in human cholangiocarcinoma cells and, the COX-2-derived prostaglandin E_2_ further activates PPAR*β*/*δ*. This positive feedback loop plays an important role for cholangiocarcinoma cell growth [54]. The role of PPAR*β*/*δ* in carcinogenesis is thus unclear and highly controversial.

## 7. HYPOTHESIS

The finding that exercise upregulates PPAR*β*/*δ* content in muscle favors a model in which the nuclear receptor plays a causal role in the increase of oxidative fiber number. Another common mark of exercise and muscle PPAR*β*/*δ* overexpression is the reduction of body fat content by a decrease in adipocyte size.

Oxidative myofiber remodeling and increase of fatty acid oxidizing actions of PPAR*β*/*δ* in skeletal muscle may give the expectations of specific agonists in metabolic syndrome by limiting substrate availability for lipid synthesis and accumulation in adipose tissue and other insulin sensitive tissues. PPAR*β*/*δ* agonists can drive the formation of oxidative myofibers and enhance physical activity endurance. Collectively, the phenotypes induced by muscle-specific PPAR*β*/*δ* overexpression, such as muscle remodeling and reduction of body fat mass, are highly reminiscent of the adaptive response to regular physical exercise. Muscle-specific PPAR*β*/*δ* agonist drugs used in combination with exercise would be a key strategy to increase physical activity-related energy expenditure and overcome the sedentary lifestyle and poor cardiorespiratory fitness.

Hypothesis is that overexpression and/or overactivity of muscle-specific PPAR*β*/*δ* by synthetic agonists strongly increase the lipid catabolic activities of skeletal muscle by upregulating genes involved in fatty acid burning and also by stimulating muscle remodeling similar to that promoted by endurance training. This adaptation may resemble that induced by endurance exercise training. In other words, synthetic activation of muscle PPAR*β*/*δ* may simulate partly the impacts of exercise training even in the absence of training itself. This strategy obviously could be beneficial to prevent metabolic disorders, such as insulin resistance, obesity, and type 2 diabetes. In addition, it is tempting to speculate that muscle-specific PPAR*β*/*δ* agonists could be expected agents for the individuals carrying metabolic risk factors who are in sedentary life style and reluctant to exercise. Finally, most likely adverse effects due to ubiquitous activation of PPAR*β*/*δ* in the long term may not be seen by the specific activation of muscle PPAR*β*/*δ*.

## 8. CONCLUSIONS

PPAR*β*/*δ* could be targeted by a specific agonist in skeletal muscle in order to prevent metabolic disorders such as insulin resistance and obesity by increasing catabolism of lipid in muscle and decreasing lipid accumulation in adipose tissue.

The importance of tissue-specific PPAR*β*/*δ* agonism is obvious because liver-specific overexpression of this transcription factor may cause glucose intolerance through the activation of PGC1*α*. However, specific activation of PPAR*β*/*δ* in skeletal muscle is very reminiscent of the adaptive response to endurance training, which also increases the levels of PPAR*β*/*δ* and PGC1*α* causing improvements in mitochondrial capacity and insulin sensitivity [25].

Mitochondrial defects are in the very heart of many age-related disorders but are also present in healthy elderly subjects, resulting in sarcopenia that is a leading cause of disability in the elderly. Fortunately, even aged muscle is still very plastic and can respond to proper stimuli by increasing its mass and strength. The ability of PPAR*β*/*δ* to stimulate mitochondrial biogenesis and oxidative function suggests that the activation of PPAR*β*/*δ* could be important for control of insulin resistance during normal aging. An exciting expectation for the future use of muscle-specific PPAR*β*/*δ* agonists might have the potential to not only slow, but also reverse mitochondrial dysfunction and thereby improve exercise performance in aging muscle.

Finally, a single bout of exercise can have a very beneficial effect on glucose metabolism and increase insulin sensitivity in sedentary subjects. Consequently, main expectation for the use of PPAR*β*/*δ* drugs with the combination of exercise would be increase physical activity to overcome the sedentary lifestyle. However, whatever the research outcomes and discoveries, probably, the “magic bullet” that promotes definite solutions to disorders in energy metabolism, triggered by excessive calorie intake and inactivity, does not exist [55].

## Figures and Tables

**Figure 1:**
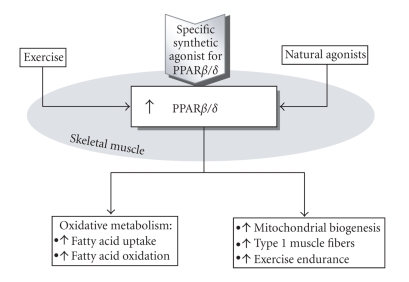
Different type of activators such as exercise and natural ligands but particularly specific synthetic agent activates PPAR*β*/*δ* in skeletal muscle and strengthens the mitochondrial apparatus and potential to oxidize lipids. More fatty acids are pulled into the more oxidative fiber made by the influence of specific agonist. Hence, PPAR*β*/*δ*-induced improvements in oxidative capacity and fat utilization in skeletal muscle could lead to metabolic improvements.
